# Supplementary material for: Resolving the full spectrum of human genome variation using Linked-Reads
Source: Genome Res. 2019 Apr;29(4):635–45. doi: 10.1101/gr.234443.118 (PMC6442396; doi:10.1101/gr.234443.118)
Supplement: Supplemental Material [file supp_gr.234443.118_Supplemental_longranger-2.2.zip › longranger-2.2.tar.bz2!/lr2.2-public/lib/stan/cmdstan/stan/doc/api/html/ast_8hpp_source.html]

Stan: src/stan/gm/ast.hpp Source File


|  |  |
| --- | --- |
|  | Stan  2.5.0  probability, sampling & optimization |


- Main Page
- Related Pages
- Namespaces
- Classes
- Files

- File List
- File Members

All Classes Namespaces Files Functions Variables Typedefs Enumerations Enumerator Friends Macros Pages

- src
- stan
- gm

ast.hpp

Go to the documentation of this file.

1 #ifndef STAN\_\_GM\_\_AST\_HPP

2 #define STAN\_\_GM\_\_AST\_HPP

3

4 #include <map>

5 #include <string>

6 #include <vector>

7 #include <map>

8 #include <set>

9

10 #include <boost/variant/recursive\_variant.hpp>

11

12 namespace stan {

13

14  namespace gm {

15

18  struct nil { };

19

20  // components of abstract syntax tree

21  struct array\_literal;

22  struct assignment;

23  struct binary\_op;

24  struct conditional\_statement;

25  struct distribution;

26  struct double\_var\_decl;

27  struct double\_literal;

28  struct expression;

29  struct for\_statement;

30  struct fun;

31  struct function\_decl\_def;

32  struct function\_decl\_defs;

33  struct identifier;

34  struct increment\_log\_prob\_statement;

35  struct index\_op;

36  struct int\_literal;

37  struct inv\_var\_decl;

38  struct matrix\_var\_decl;

39  struct no\_op\_statement;

40  struct ordered\_var\_decl;

41  struct positive\_ordered\_var\_decl;

42  struct print\_statement;

43  struct program;

44  struct range;

45  struct reject\_statement;

46  struct return\_statement;

47  struct row\_vector\_var\_decl;

48  struct sample;

49  struct simplex\_var\_decl;

50  struct integrate\_ode;

51  struct unit\_vector\_var\_decl;

52  struct statement;

53  struct statements;

54  struct unary\_op;

55  struct variable;

56  struct variable\_dims;

57  struct var\_decl;

58  struct var\_type;

59  struct vector\_var\_decl;

60  struct while\_statement;

61

62  // forward declarable enum hack (can't fwd-decl enum)

63  typedef int base\_expr\_type;

64  const int VOID\_T = 0;

65  const int INT\_T = 1;

66  const int DOUBLE\_T = 2;

67  const int VECTOR\_T = 3;

68  const int ROW\_VECTOR\_T = 4;

69  const int MATRIX\_T = 5;

70  const int ILL\_FORMED\_T = 6;

71

72  std::ostream& write\_base\_expr\_type(std::ostream& o, base\_expr\_type type);

73

74  struct expr\_type {

75  base\_expr\_type base\_type\_;

76  size\_t num\_dims\_;

77  expr\_type();

78  expr\_type(const base\_expr\_type base\_type);

79  expr\_type(const base\_expr\_type base\_type,

80  size\_t num\_dims);

81  bool operator==(const expr\_type& et) const;

82  bool operator!=(const expr\_type& et) const;

83  bool operator<(const expr\_type& et) const;

84  bool operator<=(const expr\_type& et) const;

85  bool operator>(const expr\_type& et) const;

86  bool operator>=(const expr\_type& et) const;

87  bool is\_primitive() const;

88  bool is\_primitive\_int() const;

89  bool is\_primitive\_double() const;

90  bool is\_ill\_formed() const;

91  bool is\_void() const;

92  base\_expr\_type type() const;

93  size\_t num\_dims() const;

94  };

95

96

97  std::ostream& operator<<(std::ostream& o, const expr\_type& et);

98

99  expr\_type promote\_primitive(const expr\_type& et);

100

101  expr\_type promote\_primitive(const expr\_type& et1,

102  const expr\_type& et2);

103

104  typedef std::pair<expr\_type, std::vector<expr\_type> > function\_signature\_t;

105

106  class function\_signatures {

107  public:

108  static function\_signatures& instance();

109  static void reset\_sigs();

110  void set\_user\_defined(const std::pair<std::string,function\_signature\_t>&

111  name\_sig);

112  bool is\_user\_defined(const std::pair<std::string,function\_signature\_t>&

113  name\_sig);

114  void add(const std::string& name,

115  const expr\_type& result\_type,

116  const std::vector<expr\_type>& arg\_types);

117  void add(const std::string& name,

118  const expr\_type& result\_type);

119  void add(const std::string& name,

120  const expr\_type& result\_type,

121  const expr\_type& arg\_type);

122  void add(const std::string& name,

123  const expr\_type& result\_type,

124  const expr\_type& arg\_type1,

125  const expr\_type& arg\_type2);

126  void add(const std::string& name,

127  const expr\_type& result\_type,

128  const expr\_type& arg\_type1,

129  const expr\_type& arg\_type2,

130  const expr\_type& arg\_type3);

131  void add(const std::string& name,

132  const expr\_type& result\_type,

133  const expr\_type& arg\_type1,

134  const expr\_type& arg\_type2,

135  const expr\_type& arg\_type3,

136  const expr\_type& arg\_type4);

137  void add(const std::string& name,

138  const expr\_type& result\_type,

139  const expr\_type& arg\_type1,

140  const expr\_type& arg\_type2,

141  const expr\_type& arg\_type3,

142  const expr\_type& arg\_type4,

143  const expr\_type& arg\_type5);

144  void add(const std::string& name,

145  const expr\_type& result\_type,

146  const expr\_type& arg\_type1,

147  const expr\_type& arg\_type2,

148  const expr\_type& arg\_type3,

149  const expr\_type& arg\_type4,

150  const expr\_type& arg\_type5,

151  const expr\_type& arg\_type6);

152  void add(const std::string& name,

153  const expr\_type& result\_type,

154  const expr\_type& arg\_type1,

155  const expr\_type& arg\_type2,

156  const expr\_type& arg\_type3,

157  const expr\_type& arg\_type4,

158  const expr\_type& arg\_type5,

159  const expr\_type& arg\_type6,

160  const expr\_type& arg\_type7);

161  void add\_nullary(const::std::string& name);

162  void add\_unary(const::std::string& name);

163  void add\_binary(const::std::string& name);

164  void add\_ternary(const::std::string& name);

165  void add\_quaternary(const::std::string& name);

166  int num\_promotions(const std::vector<expr\_type>& call\_args,

167  const std::vector<expr\_type>& sig\_args);

168  expr\_type get\_result\_type(const std::string& name,

169  const std::vector<expr\_type>& args,

170  std::ostream& error\_msgs);

171  int get\_signature\_matches(const std::string& name,

172  const std::vector<expr\_type>& args,

173  function\_signature\_t& signature);

174  bool is\_defined(const std::string& name,

175  const function\_signature\_t& sig);

176  std::set<std::string> key\_set() const;

177  private:

178  function\_signatures();

179  function\_signatures(const function\_signatures& fs);

180  std::map<std::string, std::vector<function\_signature\_t> > sigs\_map\_;

181  std::set<std::pair<std::string,function\_signature\_t> > user\_defined\_set\_;

182  static function\_signatures\* sigs\_; // init below outside of class

183  };

184

185  struct statements {

186  std::vector<var\_decl> local\_decl\_;

187  std::vector<statement> statements\_;

188  statements();

189  statements(const std::vector<var\_decl>& local\_decl,

190  const std::vector<statement>& stmts);

191  };

192

193

194  struct distribution {

195  std::string family\_;

196  std::vector<expression> args\_;

197  };

198

199  struct expression\_type\_vis : public boost::static\_visitor<expr\_type> {

200  expr\_type operator()(const nil& e) const;

201  expr\_type operator()(const int\_literal& e) const;

202  expr\_type operator()(const double\_literal& e) const;

203  expr\_type operator()(const array\_literal& e) const;

204  expr\_type operator()(const variable& e) const;

205  expr\_type operator()(const fun& e) const;

206  expr\_type operator()(const integrate\_ode& e) const;

207  expr\_type operator()(const index\_op& e) const;

208  expr\_type operator()(const binary\_op& e) const;

209  expr\_type operator()(const unary\_op& e) const;

210  // template <typename T> expr\_type operator()(const T& e) const;

211  };

212

213

214  struct expression;

215

216  struct expression {

217  typedef boost::variant<boost::recursive\_wrapper<nil>,

218  boost::recursive\_wrapper<int\_literal>,

219  boost::recursive\_wrapper<double\_literal>,

220  boost::recursive\_wrapper<array\_literal>,

221  boost::recursive\_wrapper<variable>,

222  boost::recursive\_wrapper<integrate\_ode>,

223  boost::recursive\_wrapper<fun>,

224  boost::recursive\_wrapper<index\_op>,

225  boost::recursive\_wrapper<binary\_op>,

226  boost::recursive\_wrapper<unary\_op> >

227  expression\_t;

228

229  expression();

230  expression(const expression& e);

231

232  // template <typename Expr> expression(const Expr& expr);

233  expression(const nil& expr);

234  expression(const int\_literal& expr);

235  expression(const double\_literal& expr);

236  expression(const array\_literal& expr);

237  expression(const variable& expr);

238  expression(const fun& expr);

239  expression(const integrate\_ode& expr);

240  expression(const index\_op& expr);

241  expression(const binary\_op& expr);

242  expression(const unary\_op& expr);

243  expression(const expression\_t& expr\_);

244

245  expr\_type expression\_type() const;

246

247  expression& operator+=(const expression& rhs);

248  expression& operator-=(const expression& rhs);

249  expression& operator\*=(const expression& rhs);

250  expression& operator/=(const expression& rhs);

251

252  expression\_t expr\_;

253  };

254

255

256  struct printable {

257  typedef boost::variant<boost::recursive\_wrapper<std::string>,

258  boost::recursive\_wrapper<expression> >

259  printable\_t;

260

261  printable();

262  printable(const expression& expression);

263  printable(const std::string& msg);

264  printable(const printable\_t& printable);

265  printable(const printable& printable);

266

267  printable\_t printable\_;

268  };

269

270  struct is\_nil\_op : public boost::static\_visitor<bool> {

271  bool operator()(const nil& x) const;

272  bool operator()(const int\_literal& x) const;

273  bool operator()(const double\_literal& x) const;

274  bool operator()(const array\_literal& x) const;

275  bool operator()(const variable& x) const;

276  bool operator()(const integrate\_ode& x) const;

277  bool operator()(const fun& x) const;

278  bool operator()(const index\_op& x) const;

279  bool operator()(const binary\_op& x) const;

280  bool operator()(const unary\_op& x) const;

281

282  // template <typename T>

283  // bool operator()(const T& x) const;

284  };

285

286  bool is\_nil(const expression& e);

287

288  struct variable\_dims {

289  std::string name\_;

290  std::vector<expression> dims\_;

291  variable\_dims();

292  variable\_dims(std::string const& name,

293  std::vector<expression> const& dims);

294  };

295

296

297  struct int\_literal {

298  int val\_;

299  expr\_type type\_;

300  int\_literal();

301  int\_literal(int val);

302  int\_literal(const int\_literal& il);

303  int\_literal& operator=(const int\_literal& il);

304  };

305

306

307  struct double\_literal {

308  double val\_;

309  expr\_type type\_;

310  double\_literal();

311  double\_literal(double val);

312  double\_literal& operator=(const double\_literal& dl);

313  };

314

315  struct array\_literal {

316  std::vector<expression> args\_;

317  expr\_type type\_;

318  array\_literal();

319  array\_literal(const std::vector<expression>& args);

320  array\_literal& operator=(const array\_literal& al);

321  };

322

323  struct variable {

324  std::string name\_;

325  expr\_type type\_;

326  variable();

327  variable(std::string name);

328  void set\_type(const base\_expr\_type& base\_type,

329  size\_t num\_dims);

330  };

331

332  struct integrate\_ode {

333  std::string system\_function\_name\_;

334  expression y0\_; // initial state

335  expression t0\_; // initial time

336  expression ts\_; // solution times

337  expression theta\_; // params

338  expression x\_; // data

339  expression x\_int\_; // integer data

340  integrate\_ode();

341  integrate\_ode(const std::string& system\_function\_name,

342  const expression& y0,

343  const expression& t0,

344  const expression& ts,

345  const expression& theta,

346  const expression& x,

347  const expression& x\_int);

348  };

349

350  struct fun {

351  std::string name\_;

352  std::vector<expression> args\_;

353  expr\_type type\_;

354  fun();

355  fun(std::string const& name,

356  std::vector<expression> const& args);

357  void infer\_type(); // FIXME: is this used anywhere?

358  };

359

360  size\_t total\_dims(const std::vector<std::vector<expression> >& dimss);

361

362  expr\_type infer\_type\_indexing(const base\_expr\_type& expr\_base\_type,

363  size\_t num\_expr\_dims,

364  size\_t num\_index\_dims);

365

366  expr\_type infer\_type\_indexing(const expression& expr,

367  size\_t num\_index\_dims);

368

369

370  struct index\_op {

371  expression expr\_;

372  std::vector<std::vector<expression> > dimss\_;

373  expr\_type type\_;

374  index\_op();

375  // vec of vec for e.g., e[1,2][3][4,5,6]

376  index\_op(const expression& expr,

377  const std::vector<std::vector<expression> >& dimss);

378  void infer\_type();

379  };

380

381

382  struct binary\_op {

383  std::string op;

384  expression left;

385  expression right;

386  expr\_type type\_;

387  binary\_op();

388  binary\_op(const expression& left,

389  const std::string& op,

390  const expression& right);

391  };

392

393  struct unary\_op {

394  char op;

395  expression subject;

396  expr\_type type\_;

397  unary\_op(char op,

398  expression const& subject);

399  };

400

401  struct range {

402  expression low\_;

403  expression high\_;

404  range();

405  range(expression const& low,

406  expression const& high);

407  bool has\_low() const;

408  bool has\_high() const;

409  };

410

411  typedef int var\_origin;

412  const int model\_name\_origin = 0;

413  const int data\_origin = 1;

414  const int transformed\_data\_origin = 2;

415  const int parameter\_origin = 3;

416  const int transformed\_parameter\_origin = 4;

417  const int derived\_origin = 5;

418  const int local\_origin = 6;

419  const int function\_argument\_origin = 7;

420  const int function\_argument\_origin\_lp = 8;

421  const int function\_argument\_origin\_rng = 9;

422  const int void\_function\_argument\_origin = 10;

423  const int void\_function\_argument\_origin\_lp = 11;

424  const int void\_function\_argument\_origin\_rng = 12;

425

426  void print\_var\_origin(std::ostream& o, const var\_origin& vo);

427

428  struct base\_var\_decl {

429  std::string name\_;

430  std::vector<expression> dims\_;

431  base\_expr\_type base\_type\_;

432  base\_var\_decl();

433  base\_var\_decl(const base\_expr\_type& base\_type);

434  base\_var\_decl(const std::string& name,

435  const std::vector<expression>& dims,

436  const base\_expr\_type& base\_type);

437  };

438

439  struct variable\_map {

440  typedef std::pair<base\_var\_decl,var\_origin> range\_t;

441

442  bool exists(const std::string& name) const;

443  base\_var\_decl get(const std::string& name) const;

444  base\_expr\_type get\_base\_type(const std::string& name) const;

445  size\_t get\_num\_dims(const std::string& name) const;

446  var\_origin get\_origin(const std::string& name) const;

447  void add(const std::string& name,

448  const base\_var\_decl& base\_decl,

449  const var\_origin& vo);

450  void remove(const std::string& name);

451

452  std::map<std::string, range\_t> map\_;

453  };

454

455  struct int\_var\_decl : public base\_var\_decl {

456  range range\_;

457  int\_var\_decl();

458  int\_var\_decl(range const& range,

459  std::string const& name,

460  std::vector<expression> const& dims);

461  };

462

463

464  struct double\_var\_decl : public base\_var\_decl {

465  range range\_;

466  double\_var\_decl();

467  double\_var\_decl(range const& range,

468  std::string const& name,

469  std::vector<expression> const& dims);

470  };

471

472  struct unit\_vector\_var\_decl : public base\_var\_decl {

473  expression K\_;

474  unit\_vector\_var\_decl();

475  unit\_vector\_var\_decl(expression const& K,

476  std::string const& name,

477  std::vector<expression> const& dims);

478  };

479

480  struct simplex\_var\_decl : public base\_var\_decl {

481  expression K\_;

482  simplex\_var\_decl();

483  simplex\_var\_decl(expression const& K,

484  std::string const& name,

485  std::vector<expression> const& dims);

486  };

487

488  struct ordered\_var\_decl : public base\_var\_decl {

489  expression K\_;

490  ordered\_var\_decl();

491  ordered\_var\_decl(expression const& K,

492  std::string const& name,

493  std::vector<expression> const& dims);

494  };

495

496  struct positive\_ordered\_var\_decl : public base\_var\_decl {

497  expression K\_;

498  positive\_ordered\_var\_decl();

499  positive\_ordered\_var\_decl(expression const& K,

500  std::string const& name,

501  std::vector<expression> const& dims);

502  };

503

504  struct vector\_var\_decl : public base\_var\_decl {

505  range range\_;

506  expression M\_;

507  vector\_var\_decl();

508  vector\_var\_decl(range const& range,

509  expression const& M,

510  std::string const& name,

511  std::vector<expression> const& dims);

512  };

513

514  struct row\_vector\_var\_decl : public base\_var\_decl {

515  range range\_;

516  expression N\_;

517  row\_vector\_var\_decl();

518  row\_vector\_var\_decl(range const& range,

519  expression const& N,

520  std::string const& name,

521  std::vector<expression> const& dims);

522  };

523

524  struct matrix\_var\_decl : public base\_var\_decl {

525  range range\_;

526  expression M\_;

527  expression N\_;

528  matrix\_var\_decl();

529  matrix\_var\_decl(range const& range,

530  expression const& M,

531  expression const& N,

532  std::string const& name,

533  std::vector<expression> const& dims);

534  };

535

536  struct cholesky\_factor\_var\_decl : public base\_var\_decl {

537  expression M\_;

538  expression N\_;

539  cholesky\_factor\_var\_decl();

540  cholesky\_factor\_var\_decl(expression const& M,

541  expression const& N,

542  std::string const& name,

543  std::vector<expression> const& dims);

544  };

545

546  struct cholesky\_corr\_var\_decl : public base\_var\_decl {

547  expression K\_;

548  cholesky\_corr\_var\_decl();

549  cholesky\_corr\_var\_decl(const expression& K,

550  const std::string& name,

551  const std::vector<expression>& dims);

552  };

553

554  struct cov\_matrix\_var\_decl : public base\_var\_decl {

555  expression K\_;

556  cov\_matrix\_var\_decl();

557  cov\_matrix\_var\_decl(expression const& K,

558  std::string const& name,

559  std::vector<expression> const& dims);

560  };

561

562

563  struct corr\_matrix\_var\_decl : public base\_var\_decl {

564  expression K\_;

565  corr\_matrix\_var\_decl();

566  corr\_matrix\_var\_decl(expression const& K,

567  std::string const& name,

568  std::vector<expression> const& dims);

569  };

570

571  struct name\_vis : public boost::static\_visitor<std::string> {

572  name\_vis();

573  std::string operator()(const nil& x) const;

574  std::string operator()(const int\_var\_decl& x) const;

575  std::string operator()(const double\_var\_decl& x) const;

576  std::string operator()(const vector\_var\_decl& x) const;

577  std::string operator()(const row\_vector\_var\_decl& x) const;

578  std::string operator()(const matrix\_var\_decl& x) const;

579  std::string operator()(const simplex\_var\_decl& x) const;

580  std::string operator()(const unit\_vector\_var\_decl& x) const;

581  std::string operator()(const ordered\_var\_decl& x) const;

582  std::string operator()(const positive\_ordered\_var\_decl& x) const;

583  std::string operator()(const cholesky\_factor\_var\_decl& x) const;

584  std::string operator()(const cholesky\_corr\_var\_decl& x) const;

585  std::string operator()(const cov\_matrix\_var\_decl& x) const;

586  std::string operator()(const corr\_matrix\_var\_decl& x) const;

587  };

588

589

590

591

592  struct var\_decl {

593  typedef boost::variant<boost::recursive\_wrapper<nil>,

594  boost::recursive\_wrapper<int\_var\_decl>,

595  boost::recursive\_wrapper<double\_var\_decl>,

596  boost::recursive\_wrapper<vector\_var\_decl>,

597  boost::recursive\_wrapper<row\_vector\_var\_decl>,

598  boost::recursive\_wrapper<matrix\_var\_decl>,

599  boost::recursive\_wrapper<simplex\_var\_decl>,

600  boost::recursive\_wrapper<unit\_vector\_var\_decl>,

601  boost::recursive\_wrapper<ordered\_var\_decl>,

602  boost::recursive\_wrapper<positive\_ordered\_var\_decl>,

603  boost::recursive\_wrapper<cholesky\_factor\_var\_decl>,

604  boost::recursive\_wrapper<cholesky\_corr\_var\_decl>,

605  boost::recursive\_wrapper<cov\_matrix\_var\_decl>,

606  boost::recursive\_wrapper<corr\_matrix\_var\_decl> >

607  var\_decl\_t;

608

609  var\_decl\_t decl\_;

610

611  var\_decl();

612

613  // template <typename Decl>

614  // var\_decl(Decl const& decl);

615  var\_decl(const var\_decl\_t& decl);

616  var\_decl(const nil& decl);

617  var\_decl(const int\_var\_decl& decl);

618  var\_decl(const double\_var\_decl& decl);

619  var\_decl(const vector\_var\_decl& decl);

620  var\_decl(const row\_vector\_var\_decl& decl);

621  var\_decl(const matrix\_var\_decl& decl);

622  var\_decl(const simplex\_var\_decl& decl);

623  var\_decl(const unit\_vector\_var\_decl& decl);

624  var\_decl(const ordered\_var\_decl& decl);

625  var\_decl(const positive\_ordered\_var\_decl& decl);

626  var\_decl(const cholesky\_factor\_var\_decl& decl);

627  var\_decl(const cholesky\_corr\_var\_decl& decl);

628  var\_decl(const cov\_matrix\_var\_decl& decl);

629  var\_decl(const corr\_matrix\_var\_decl& decl);

630

631  std::string name() const;

632  };

633

634  struct statement {

635  typedef boost::variant<boost::recursive\_wrapper<nil>,

636  boost::recursive\_wrapper<assignment>,

637  boost::recursive\_wrapper<sample>,

638  boost::recursive\_wrapper<increment\_log\_prob\_statement>,

639  boost::recursive\_wrapper<expression>, // dummy now

640  boost::recursive\_wrapper<statements>,

641  boost::recursive\_wrapper<for\_statement>,

642  boost::recursive\_wrapper<conditional\_statement>,

643  boost::recursive\_wrapper<while\_statement>,

644  boost::recursive\_wrapper<print\_statement>,

645  boost::recursive\_wrapper<reject\_statement>,

646  boost::recursive\_wrapper<return\_statement>,

647  boost::recursive\_wrapper<no\_op\_statement> >

648  statement\_t;

649

650  statement\_t statement\_;

651

652  statement();

653  statement(const statement\_t& st);

654  statement(const nil& st);

655  statement(const assignment& st);

656  statement(const sample& st);

657  statement(const increment\_log\_prob\_statement& st);

658  statement(const expression& st);

659  statement(const statements& st);

660  statement(const for\_statement& st);

661  statement(const conditional\_statement& st);

662  statement(const while\_statement& st);

663  statement(const print\_statement& st);

664  statement(const reject\_statement& st);

665  statement(const no\_op\_statement& st);

666  statement(const return\_statement& st);

667

668  bool is\_no\_op\_statement() const;

669  };

670

671  struct is\_no\_op\_statement\_vis : public boost::static\_visitor<bool> {

672  bool operator()(const nil& st) const;

673  bool operator()(const assignment& st) const;

674  bool operator()(const sample& st) const;

675  bool operator()(const increment\_log\_prob\_statement& t) const;

676  bool operator()(const expression& st) const;

677  bool operator()(const statements& st) const;

678  bool operator()(const for\_statement& st) const;

679  bool operator()(const conditional\_statement& st) const;

680  bool operator()(const while\_statement& st) const;

681  bool operator()(const print\_statement& st) const;

682  bool operator()(const reject\_statement& st) const;

683  bool operator()(const no\_op\_statement& st) const;

684  bool operator()(const return\_statement& st) const;

685  };

686

687

688  struct returns\_type\_vis : public boost::static\_visitor<bool> {

689  expr\_type return\_type\_;

690  std::ostream& error\_msgs\_;

691  returns\_type\_vis(const expr\_type& return\_type,

692  std::ostream& error\_msgs);

693  bool operator()(const nil& st) const;

694  bool operator()(const assignment& st) const;

695  bool operator()(const sample& st) const;

696  bool operator()(const increment\_log\_prob\_statement& t) const;

697  bool operator()(const expression& st) const;

698  bool operator()(const statements& st) const;

699  bool operator()(const for\_statement& st) const;

700  bool operator()(const conditional\_statement& st) const;

701  bool operator()(const while\_statement& st) const;

702  bool operator()(const print\_statement& st) const;

703  bool operator()(const reject\_statement& st) const;

704  bool operator()(const no\_op\_statement& st) const;

705  bool operator()(const return\_statement& st) const;

706  };

707

708  bool returns\_type(const expr\_type& return\_type,

709  const statement& statement,

710  std::ostream& error\_msgs);

711

712  struct increment\_log\_prob\_statement {

713  expression log\_prob\_;

714  increment\_log\_prob\_statement();

715  increment\_log\_prob\_statement(const expression& log\_prob);

716  };

717

718  struct for\_statement {

719  std::string variable\_;

720  range range\_;

721  statement statement\_;

722  for\_statement();

723  for\_statement(std::string& variable,

724  range& range,

725  statement& stmt);

726  };

727

728  // bodies may be 1 longer than conditions due to else

729  struct conditional\_statement {

730  std::vector<expression> conditions\_;

731  std::vector<statement> bodies\_;

732  conditional\_statement();

733  conditional\_statement(const std::vector<expression>& conditions,

734  const std::vector<statement>& statements);

735  };

736

737  struct while\_statement {

738  expression condition\_;

739  statement body\_;

740  while\_statement();

741  while\_statement(const expression& condition,

742  const statement& body);

743  };

744

745  struct print\_statement {

746  std::vector<printable> printables\_;

747  print\_statement();

748  print\_statement(const std::vector<printable>& printables);

749  };

750

751  struct reject\_statement {

752  std::vector<printable> printables\_;

753  reject\_statement();

754  reject\_statement(const std::vector<printable>& printables);

755  };

756

757  struct return\_statement {

758  expression return\_value\_;

759  return\_statement();

760  return\_statement(const expression& expr);

761  };

762

763  struct no\_op\_statement {

764  // no op, no data

765  };

766

767  struct arg\_decl {

768  expr\_type arg\_type\_;

769  std::string name\_;

770  arg\_decl();

771  arg\_decl(const expr\_type& arg\_type,

772  const std::string& name);

773  base\_var\_decl base\_variable\_declaration();

774  };

775

776  struct function\_decl\_def {

777  function\_decl\_def();

778  function\_decl\_def(const expr\_type& return\_type,

779  const std::string& name,

780  const std::vector<arg\_decl>& arg\_decls,

781  const statement& body);

782  expr\_type return\_type\_;

783  std::string name\_;

784  std::vector<arg\_decl> arg\_decls\_;

785  statement body\_;

786  };

787

788  struct function\_decl\_defs {

789  function\_decl\_defs();

790  function\_decl\_defs(const std::vector<function\_decl\_def>& decl\_defs);

791  std::vector<function\_decl\_def> decl\_defs\_;

792  };

793

794

795  struct program {

796  std::vector<function\_decl\_def> function\_decl\_defs\_;

797  std::vector<var\_decl> data\_decl\_;

798  std::pair<std::vector<var\_decl>,std::vector<statement> >

799  derived\_data\_decl\_;

800  std::vector<var\_decl> parameter\_decl\_;

801  std::pair<std::vector<var\_decl>,std::vector<statement> >

802  derived\_decl\_;

803  statement statement\_;

804  std::pair<std::vector<var\_decl>,std::vector<statement> > generated\_decl\_;

805  program();

806  program(const std::vector<function\_decl\_def>& function\_decl\_defs,

807  const std::vector<var\_decl>& data\_decl,

808  const std::pair<std::vector<var\_decl>,

809  std::vector<statement> >& derived\_data\_decl,

810  const std::vector<var\_decl>& parameter\_decl,

811  const std::pair<std::vector<var\_decl>,

812  std::vector<statement> >& derived\_decl,

813  const statement& st,

814  const std::pair<std::vector<var\_decl>,

815  std::vector<statement> >& generated\_decl);

816  };

817

818  struct sample {

819  expression expr\_;

820  distribution dist\_;

821  range truncation\_;

822  sample();

823  sample(expression& e,

824  distribution& dist);

825  bool is\_ill\_formed() const;

826  };

827

828  struct assignment {

829  variable\_dims var\_dims\_; // lhs\_var[dim0,...,dimN-1]

830  expression expr\_; // = rhs

831  base\_var\_decl var\_type\_; // type of lhs\_var

832  assignment();

833  assignment(variable\_dims& var\_dims,

834  expression& expr);

835  };

836

837  // FIXME: is this next dependency necessary?

838  // from generator.hpp

839  void generate\_expression(const expression& e, std::ostream& o);

840

841  bool has\_rng\_suffix(const std::string& s);

842  bool has\_lp\_suffix(const std::string& s);

843  bool is\_user\_defined(const std::string& name,

844  const std::vector<expression>& args);

845  bool is\_user\_defined\_prob\_function(const std::string& name,

846  const expression& variate,

847  const std::vector<expression>& params);

848  bool is\_user\_defined(const fun& fx);

849

850  struct contains\_var : public boost::static\_visitor<bool> {

851  const variable\_map& var\_map\_;

852  contains\_var(const variable\_map& var\_map);

853  bool operator()(const nil& e) const;

854  bool operator()(const int\_literal& e) const;

855  bool operator()(const double\_literal& e) const;

856  bool operator()(const array\_literal& e) const;

857  bool operator()(const variable& e) const;

858  bool operator()(const integrate\_ode& e) const;

859  bool operator()(const fun& e) const;

860  bool operator()(const index\_op& e) const;

861  bool operator()(const binary\_op& e) const;

862  bool operator()(const unary\_op& e) const;

863  };

864

865  bool has\_var(const expression& e,

866  const variable\_map& var\_map);

867

868

869  struct contains\_nonparam\_var : public boost::static\_visitor<bool> {

870  const variable\_map& var\_map\_;

871  contains\_nonparam\_var(const variable\_map& var\_map);

872  bool operator()(const nil& e) const;

873  bool operator()(const int\_literal& e) const;

874  bool operator()(const double\_literal& e) const;

875  bool operator()(const array\_literal& e) const;

876  bool operator()(const variable& e) const;

877  bool operator()(const integrate\_ode& e) const;

878  bool operator()(const fun& e) const;

879  bool operator()(const index\_op& e) const;

880  bool operator()(const binary\_op& e) const;

881  bool operator()(const unary\_op& e) const;

882  };

883

884  bool has\_non\_param\_var(const expression& e,

885  const variable\_map& var\_map);

886

887  bool is\_assignable(const expr\_type& l\_type,

888  const expr\_type& r\_type,

889  const std::string& failure\_message,

890  std::ostream& error\_msgs);

891

892

893  bool ends\_with(const std::string& suffix,

894  const std::string& s);

895

896  }

897 }

898

899 #endif

stan::gm::var\_decl::var\_decl

var\_decl()

**Definition:** ast\_def.cpp:1208

stan::gm::expr\_type::operator==

bool operator==(const expr\_type &et) const

**Definition:** ast\_def.cpp:66

stan::gm::distribution::family\_

std::string family\_

**Definition:** ast.hpp:195

stan::gm::integrate\_ode::ts\_

expression ts\_

**Definition:** ast.hpp:336

stan::gm::row\_vector\_var\_decl::range\_

range range\_

**Definition:** ast.hpp:515

stan::gm::nil

Placeholder struct for boost::variant default ctors.

**Definition:** ast.hpp:18

stan::gm::expression::operator/=

expression & operator/=(const expression &rhs)

**Definition:** ast\_def.cpp:1401

stan::gm::variable\_dims

**Definition:** ast.hpp:288

stan::gm::increment\_log\_prob\_statement::log\_prob\_

expression log\_prob\_

**Definition:** ast.hpp:713

stan::gm::matrix\_var\_decl::range\_

range range\_

**Definition:** ast.hpp:525

stan::gm::statement

**Definition:** ast.hpp:634

stan::gm::generate\_expression

void generate\_expression(const expression &e, std::ostream &o)

**Definition:** generator.hpp:249

stan::gm::double\_var\_decl::range\_

range range\_

**Definition:** ast.hpp:465

stan::gm::function\_signatures::is\_user\_defined

bool is\_user\_defined(const std::pair< std::string, function\_signature\_t > &name\_sig)

**Definition:** ast\_def.cpp:157

stan::gm::statement::is\_no\_op\_statement

bool is\_no\_op\_statement() const

**Definition:** ast\_def.cpp:1286

stan::gm::function\_signatures::add\_unary

void add\_unary(const ::std::string &name)

**Definition:** ast\_def.cpp:276

stan::gm::corr\_matrix\_var\_decl

**Definition:** ast.hpp:563

stan::gm::integrate\_ode::x\_

expression x\_

**Definition:** ast.hpp:338

stan::gm::ends\_with

bool ends\_with(const std::string &suffix, const std::string &s)

**Definition:** ast\_def.cpp:1477

stan::gm::program::derived\_data\_decl\_

std::pair< std::vector< var\_decl >, std::vector< statement > > derived\_data\_decl\_

**Definition:** ast.hpp:799

stan::gm::cov\_matrix\_var\_decl::cov\_matrix\_var\_decl

cov\_matrix\_var\_decl()

**Definition:** ast\_def.cpp:1140

stan::gm::conditional\_statement::conditions\_

std::vector< expression > conditions\_

**Definition:** ast.hpp:730

stan::gm::row\_vector\_var\_decl::N\_

expression N\_

**Definition:** ast.hpp:516

stan::gm::fun::name\_

std::string name\_

**Definition:** ast.hpp:351

stan::gm::function\_signatures::add

void add(const std::string &name, const expr\_type &result\_type, const std::vector< expr\_type > &arg\_types)

**Definition:** ast\_def.cpp:171

stan::gm::base\_var\_decl::dims\_

std::vector< expression > dims\_

**Definition:** ast.hpp:430

stan::gm::binary\_op::right

expression right

**Definition:** ast.hpp:385

stan::gm::expr\_type::base\_type\_

base\_expr\_type base\_type\_

**Definition:** ast.hpp:75

stan::gm::data\_origin

const int data\_origin

**Definition:** ast.hpp:413

stan::gm::function\_signatures::add\_nullary

void add\_nullary(const ::std::string &name)

**Definition:** ast\_def.cpp:273

stan::gm::increment\_log\_prob\_statement::increment\_log\_prob\_statement

increment\_log\_prob\_statement()

**Definition:** ast\_def.cpp:1291

stan::gm::assignment::var\_dims\_

variable\_dims var\_dims\_

**Definition:** ast.hpp:829

stan::gm::double\_var\_decl

**Definition:** ast.hpp:464

stan::gm::parameter\_origin

const int parameter\_origin

**Definition:** ast.hpp:415

stan::gm::print\_statement::print\_statement

print\_statement()

**Definition:** ast\_def.cpp:1329

stan::gm::corr\_matrix\_var\_decl::K\_

expression K\_

**Definition:** ast.hpp:564

stan::gm::variable\_map::add

void add(const std::string &name, const base\_var\_decl &base\_decl, const var\_origin &vo)

**Definition:** ast\_def.cpp:1007

stan::gm::fun::type\_

expr\_type type\_

**Definition:** ast.hpp:353

stan::gm::is\_user\_defined\_prob\_function

bool is\_user\_defined\_prob\_function(const std::string &name, const expression &variate, const std::vector< expression > &params)

**Definition:** ast\_def.cpp:1439

stan::gm::program::statement\_

statement statement\_

**Definition:** ast.hpp:803

stan::gm::cholesky\_corr\_var\_decl::K\_

expression K\_

**Definition:** ast.hpp:547

stan::gm::function\_signatures::is\_defined

bool is\_defined(const std::string &name, const function\_signature\_t &sig)

**Definition:** ast\_def.cpp:163

stan::gm::DOUBLE\_T

const int DOUBLE\_T

**Definition:** ast.hpp:66

stan::gm::arg\_decl::name\_

std::string name\_

**Definition:** ast.hpp:769

stan::gm::row\_vector\_var\_decl

**Definition:** ast.hpp:514

stan::gm::function\_signatures::instance

static function\_signatures & instance()

**Definition:** ast\_def.cpp:143

stan::gm::unary\_op

**Definition:** ast.hpp:393

stan::gm::integrate\_ode::y0\_

expression y0\_

**Definition:** ast.hpp:334

stan::gm::fun::infer\_type

void infer\_type()

**Definition:** ast\_def.cpp:860

stan::gm::array\_literal

**Definition:** ast.hpp:315

stan::gm::local\_origin

const int local\_origin

**Definition:** ast.hpp:418

stan::gm::distribution

**Definition:** ast.hpp:194

stan::gm::assignment::expr\_

expression expr\_

**Definition:** ast.hpp:830

stan::gm::statements

**Definition:** ast.hpp:185

stan::gm::range

**Definition:** ast.hpp:401

stan::gm::function\_decl\_defs::function\_decl\_defs

function\_decl\_defs()

**Definition:** ast\_def.cpp:422

stan::gm::variable\_map::map\_

std::map< std::string, range\_t > map\_

**Definition:** ast.hpp:452

stan::gm::printable::printable\_t

boost::variant< boost::recursive\_wrapper< std::string >, boost::recursive\_wrapper< expression > > printable\_t

**Definition:** ast.hpp:259

stan::gm::ordered\_var\_decl::K\_

expression K\_

**Definition:** ast.hpp:489

stan::gm::unary\_op::type\_

expr\_type type\_

**Definition:** ast.hpp:396

stan::gm::conditional\_statement

**Definition:** ast.hpp:729

stan::gm::assignment

**Definition:** ast.hpp:828

stan::gm::integrate\_ode::x\_int\_

expression x\_int\_

**Definition:** ast.hpp:339

stan::gm::ordered\_var\_decl

**Definition:** ast.hpp:488

stan::gm::index\_op::dimss\_

std::vector< std::vector< expression > > dimss\_

**Definition:** ast.hpp:372

stan::gm::expr\_type::operator>=

bool operator>=(const expr\_type &et) const

**Definition:** ast\_def.cpp:88

stan::gm::variable::variable

variable()

**Definition:** ast\_def.cpp:827

stan::gm::expr\_type::is\_primitive\_int

bool is\_primitive\_int() const

**Definition:** ast\_def.cpp:97

stan::gm::binary\_op::type\_

expr\_type type\_

**Definition:** ast.hpp:386

stan::gm::function\_signatures::set\_user\_defined

void set\_user\_defined(const std::pair< std::string, function\_signature\_t > &name\_sig)

**Definition:** ast\_def.cpp:150

stan::gm::int\_literal

**Definition:** ast.hpp:297

stan::gm::range::has\_low

bool has\_low() const

**Definition:** ast\_def.cpp:937

stan::gm::range::high\_

expression high\_

**Definition:** ast.hpp:403

stan::gm::expr\_type::is\_primitive\_double

bool is\_primitive\_double() const

**Definition:** ast\_def.cpp:101

stan::gm::arg\_decl::arg\_decl

arg\_decl()

**Definition:** ast\_def.cpp:397

stan::gm::integrate\_ode::integrate\_ode

integrate\_ode()

**Definition:** ast\_def.cpp:835

stan::gm::matrix\_var\_decl::matrix\_var\_decl

matrix\_var\_decl()

**Definition:** ast\_def.cpp:1105

stan::gm::program::generated\_decl\_

std::pair< std::vector< var\_decl >, std::vector< statement > > generated\_decl\_

**Definition:** ast.hpp:804

stan::gm::vector\_var\_decl::range\_

range range\_

**Definition:** ast.hpp:505

stan::gm::ROW\_VECTOR\_T

const int ROW\_VECTOR\_T

**Definition:** ast.hpp:68

stan::gm::program::data\_decl\_

std::vector< var\_decl > data\_decl\_

**Definition:** ast.hpp:797

stan::gm::print\_var\_origin

void print\_var\_origin(std::ostream &o, const var\_origin &vo)

**Definition:** ast\_def.cpp:944

stan::gm::returns\_type\_vis::return\_type\_

expr\_type return\_type\_

**Definition:** ast.hpp:689

stan::gm::function\_signatures::reset\_sigs

static void reset\_sigs()

**Definition:** ast\_def.cpp:138

stan::gm::function\_decl\_def

**Definition:** ast.hpp:776

stan::gm::function\_argument\_origin

const int function\_argument\_origin

**Definition:** ast.hpp:419

stan::gm::is\_user\_defined

bool is\_user\_defined(const std::string &name, const std::vector< expression > &args)

**Definition:** ast\_def.cpp:1423

stan::gm::expression::operator-=

expression & operator-=(const expression &rhs)

**Definition:** ast\_def.cpp:1391

stan::gm::no\_op\_statement

**Definition:** ast.hpp:763

stan::gm::is\_nil\_op

**Definition:** ast.hpp:270

stan::gm::expression\_type\_vis

**Definition:** ast.hpp:199

stan::gm::variable\_dims::variable\_dims

variable\_dims()

**Definition:** ast\_def.cpp:773

stan::return\_type

Metaprogram to calculate the base scalar return type resulting from promoting all the scalar types of...

**Definition:** traits.hpp:398

stan::gm::cov\_matrix\_var\_decl::K\_

expression K\_

**Definition:** ast.hpp:555

stan::gm::program

**Definition:** ast.hpp:795

stan::gm::function\_signatures::get\_signature\_matches

int get\_signature\_matches(const std::string &name, const std::vector< expr\_type > &args, function\_signature\_t &signature)

**Definition:** ast\_def.cpp:307

stan::gm::integrate\_ode

**Definition:** ast.hpp:332

stan::gm::expression::operator\*=

expression & operator\*=(const expression &rhs)

**Definition:** ast\_def.cpp:1396

stan::gm::total\_dims

size\_t total\_dims(const std::vector< std::vector< expression > > &dimss)

**Definition:** ast\_def.cpp:865

stan::gm::simplex\_var\_decl::K\_

expression K\_

**Definition:** ast.hpp:481

stan::gm::void\_function\_argument\_origin\_rng

const int void\_function\_argument\_origin\_rng

**Definition:** ast.hpp:424

stan::gm::array\_literal::array\_literal

array\_literal()

**Definition:** ast\_def.cpp:813

stan::gm::cov\_matrix\_var\_decl

**Definition:** ast.hpp:554

stan::gm::expression::expression

expression()

**Definition:** ast\_def.cpp:564

stan::gm::return\_statement::return\_value\_

expression return\_value\_

**Definition:** ast.hpp:758

stan::gm::function\_argument\_origin\_rng

const int function\_argument\_origin\_rng

**Definition:** ast.hpp:421

stan::gm::cholesky\_factor\_var\_decl::M\_

expression M\_

**Definition:** ast.hpp:537

stan::gm::has\_rng\_suffix

bool has\_rng\_suffix(const std::string &s)

**Definition:** ast\_def.cpp:1406

stan::gm::fun::args\_

std::vector< expression > args\_

**Definition:** ast.hpp:352

stan::gm::function\_signatures

**Definition:** ast.hpp:106

stan::gm::function\_signatures::add\_binary

void add\_binary(const ::std::string &name)

**Definition:** ast\_def.cpp:279

stan::gm::for\_statement

**Definition:** ast.hpp:718

stan::gm::contains\_nonparam\_var

**Definition:** ast.hpp:869

stan::gm::ordered\_var\_decl::ordered\_var\_decl

ordered\_var\_decl()

**Definition:** ast\_def.cpp:1062

stan::gm::index\_op::index\_op

index\_op()

**Definition:** ast\_def.cpp:900

stan::gm::vector\_var\_decl::M\_

expression M\_

**Definition:** ast.hpp:506

stan::gm::increment\_log\_prob\_statement

**Definition:** ast.hpp:712

stan::gm::printable

**Definition:** ast.hpp:256

stan::gm::print\_statement

**Definition:** ast.hpp:745

stan::gm::contains\_var::operator()

bool operator()(const nil &e) const

**Definition:** ast\_def.cpp:600

stan::gm::cholesky\_factor\_var\_decl::N\_

expression N\_

**Definition:** ast.hpp:538

stan::gm::sample::is\_ill\_formed

bool is\_ill\_formed() const

**Definition:** ast\_def.cpp:1368

stan::gm::sample::expr\_

expression expr\_

**Definition:** ast.hpp:819

stan::gm::simplex\_var\_decl::simplex\_var\_decl

simplex\_var\_decl()

**Definition:** ast\_def.cpp:1051

stan::gm::variable\_map::get\_num\_dims

size\_t get\_num\_dims(const std::string &name) const

**Definition:** ast\_def.cpp:999

stan::gm::sample::sample

sample()

**Definition:** ast\_def.cpp:1361

stan::gm::array\_literal::args\_

std::vector< expression > args\_

**Definition:** ast.hpp:316

stan::gm::double\_literal

**Definition:** ast.hpp:307

stan::gm::base\_var\_decl::base\_type\_

base\_expr\_type base\_type\_

**Definition:** ast.hpp:431

stan::gm::double\_literal::double\_literal

double\_literal()

**Definition:** ast\_def.cpp:799

stan::gm::expression::expr\_

expression\_t expr\_

**Definition:** ast.hpp:252

stan::gm::while\_statement::body\_

statement body\_

**Definition:** ast.hpp:739

stan::gm::returns\_type\_vis::error\_msgs\_

std::ostream & error\_msgs\_

**Definition:** ast.hpp:690

stan::gm::program::parameter\_decl\_

std::vector< var\_decl > parameter\_decl\_

**Definition:** ast.hpp:800

stan::gm::var\_decl

**Definition:** ast.hpp:592

stan::gm::expr\_type::num\_dims\_

size\_t num\_dims\_

**Definition:** ast.hpp:76

stan::gm::expr\_type::type

base\_expr\_type type() const

**Definition:** ast\_def.cpp:111

stan::gm::has\_non\_param\_var

bool has\_non\_param\_var(const expression &e, const variable\_map &var\_map)

**Definition:** ast\_def.cpp:748

stan::gm::unary\_op::subject

expression subject

**Definition:** ast.hpp:395

stan::gm::program::program

program()

**Definition:** ast\_def.cpp:1341

stan::gm::derived\_origin

const int derived\_origin

**Definition:** ast.hpp:417

stan::gm::operator<<

std::ostream & operator<<(std::ostream &o, const expr\_type &et)

**Definition:** ast\_def.cpp:118

stan::gm::base\_var\_decl

**Definition:** ast.hpp:428

stan::gm::conditional\_statement::bodies\_

std::vector< statement > bodies\_

**Definition:** ast.hpp:731

stan::gm::while\_statement::condition\_

expression condition\_

**Definition:** ast.hpp:738

stan::gm::integrate\_ode::t0\_

expression t0\_

**Definition:** ast.hpp:335

stan::gm::cholesky\_factor\_var\_decl

**Definition:** ast.hpp:536

stan::gm::int\_literal::type\_

expr\_type type\_

**Definition:** ast.hpp:299

stan::gm::positive\_ordered\_var\_decl

**Definition:** ast.hpp:496

stan::gm::contains\_var

**Definition:** ast.hpp:850

stan::gm::int\_var\_decl::range\_

range range\_

**Definition:** ast.hpp:456

stan::gm::matrix\_var\_decl::N\_

expression N\_

**Definition:** ast.hpp:527

stan::gm::integrate\_ode::theta\_

expression theta\_

**Definition:** ast.hpp:337

stan::gm::function\_signatures::num\_promotions

int num\_promotions(const std::vector< expr\_type > &call\_args, const std::vector< expr\_type > &sig\_args)

**Definition:** ast\_def.cpp:288

stan::gm::row\_vector\_var\_decl::row\_vector\_var\_decl

row\_vector\_var\_decl()

**Definition:** ast\_def.cpp:1095

stan::gm::base\_var\_decl::base\_var\_decl

base\_var\_decl()

**Definition:** ast\_def.cpp:976

stan::gm::variable\_map

**Definition:** ast.hpp:439

stan::gm::VECTOR\_T

const int VECTOR\_T

**Definition:** ast.hpp:67

stan::gm::program::derived\_decl\_

std::pair< std::vector< var\_decl >, std::vector< statement > > derived\_decl\_

**Definition:** ast.hpp:802

stan::gm::double\_literal::val\_

double val\_

**Definition:** ast.hpp:308

stan::gm::MATRIX\_T

const int MATRIX\_T

**Definition:** ast.hpp:69

stan::gm::promote\_primitive

expr\_type promote\_primitive(const expr\_type &et)

**Definition:** ast\_def.cpp:125

stan::gm::function\_decl\_def::function\_decl\_def

function\_decl\_def()

**Definition:** ast\_def.cpp:410

stan::gm::index\_op::type\_

expr\_type type\_

**Definition:** ast.hpp:373

stan::math::dims

void dims(const T &x, std::vector< int > &result)

**Definition:** dims.hpp:13

stan::gm::returns\_type\_vis::returns\_type\_vis

returns\_type\_vis(const expr\_type &return\_type, std::ostream &error\_msgs)

**Definition:** ast\_def.cpp:428

stan::gm::cholesky\_corr\_var\_decl::cholesky\_corr\_var\_decl

cholesky\_corr\_var\_decl()

**Definition:** ast\_def.cpp:1130

stan::gm::range::low\_

expression low\_

**Definition:** ast.hpp:402

stan::gm::expr\_type

**Definition:** ast.hpp:74

stan::gm::var\_decl::name

std::string name() const

**Definition:** ast\_def.cpp:1224

stan::gm::expression::expression\_t

boost::variant< boost::recursive\_wrapper< nil >, boost::recursive\_wrapper< int\_literal >, boost::recursive\_wrapper< double\_literal >, boost::recursive\_wrapper< array\_literal >, boost::recursive\_wrapper< variable >, boost::recursive\_wrapper< integrate\_ode >, boost::recursive\_wrapper< fun >, boost::recursive\_wrapper< index\_op >, boost::recursive\_wrapper< binary\_op >, boost::recursive\_wrapper< unary\_op > > expression\_t

**Definition:** ast.hpp:227

stan::gm::printable::printable\_

printable\_t printable\_

**Definition:** ast.hpp:267

stan::gm::vector\_var\_decl::vector\_var\_decl

vector\_var\_decl()

**Definition:** ast\_def.cpp:1084

stan::gm::int\_literal::int\_literal

int\_literal()

**Definition:** ast\_def.cpp:781

stan::gm::variable\_dims::dims\_

std::vector< expression > dims\_

**Definition:** ast.hpp:290

stan::gm::contains\_nonparam\_var::contains\_nonparam\_var

contains\_nonparam\_var(const variable\_map &var\_map)

**Definition:** ast\_def.cpp:681

stan::gm::unit\_vector\_var\_decl::K\_

expression K\_

**Definition:** ast.hpp:473

stan::gm::void\_function\_argument\_origin\_lp

const int void\_function\_argument\_origin\_lp

**Definition:** ast.hpp:423

stan::gm::expr\_type::num\_dims

size\_t num\_dims() const

**Definition:** ast\_def.cpp:114

stan::gm::for\_statement::statement\_

statement statement\_

**Definition:** ast.hpp:721

stan::gm::variable\_dims::name\_

std::string name\_

**Definition:** ast.hpp:289

stan::gm::for\_statement::range\_

range range\_

**Definition:** ast.hpp:720

stan::gm::matrix\_var\_decl

**Definition:** ast.hpp:524

stan::gm::has\_var

bool has\_var(const expression &e, const variable\_map &var\_map)

**Definition:** ast\_def.cpp:675

stan::gm::binary\_op::left

expression left

**Definition:** ast.hpp:384

stan::gm::double\_var\_decl::double\_var\_decl

double\_var\_decl()

**Definition:** ast\_def.cpp:1029

stan::gm::expression

**Definition:** ast.hpp:216

stan::gm::index\_op::expr\_

expression expr\_

**Definition:** ast.hpp:371

stan::gm::function\_decl\_def::arg\_decls\_

std::vector< arg\_decl > arg\_decls\_

**Definition:** ast.hpp:784

stan::gm::binary\_op::op

std::string op

**Definition:** ast.hpp:383

stan::gm::function\_decl\_defs

**Definition:** ast.hpp:788

stan::gm::variable::type\_

expr\_type type\_

**Definition:** ast.hpp:325

stan::gm::base\_var\_decl::name\_

std::string name\_

**Definition:** ast.hpp:429

stan::gm::void\_function\_argument\_origin

const int void\_function\_argument\_origin

**Definition:** ast.hpp:422

stan::gm::simplex\_var\_decl

**Definition:** ast.hpp:480

stan::gm::expr\_type::expr\_type

expr\_type()

**Definition:** ast\_def.cpp:53

stan::gm::function\_decl\_def::name\_

std::string name\_

**Definition:** ast.hpp:783

stan::gm::assignment::assignment

assignment()

**Definition:** ast\_def.cpp:1378

stan::gm::sample

**Definition:** ast.hpp:818

stan::gm::function\_signatures::add\_quaternary

void add\_quaternary(const ::std::string &name)

**Definition:** ast\_def.cpp:285

stan::gm::cholesky\_corr\_var\_decl

**Definition:** ast.hpp:546

stan::gm::reject\_statement

**Definition:** ast.hpp:751

stan::gm::model\_name\_origin

const int model\_name\_origin

**Definition:** ast.hpp:412

stan::gm::has\_lp\_suffix

bool has\_lp\_suffix(const std::string &s)

**Definition:** ast\_def.cpp:1415

stan::gm::name\_vis

**Definition:** ast.hpp:571

stan::gm::statement::statement\_t

boost::variant< boost::recursive\_wrapper< nil >, boost::recursive\_wrapper< assignment >, boost::recursive\_wrapper< sample >, boost::recursive\_wrapper< increment\_log\_prob\_statement >, boost::recursive\_wrapper< expression >, boost::recursive\_wrapper< statements >, boost::recursive\_wrapper< for\_statement >, boost::recursive\_wrapper< conditional\_statement >, boost::recursive\_wrapper< while\_statement >, boost::recursive\_wrapper< print\_statement >, boost::recursive\_wrapper< reject\_statement >, boost::recursive\_wrapper< return\_statement >, boost::recursive\_wrapper< no\_op\_statement > > statement\_t

**Definition:** ast.hpp:648

stan::gm::array\_literal::operator=

array\_literal & operator=(const array\_literal &al)

**Definition:** ast\_def.cpp:821

stan::gm::function\_signatures::key\_set

std::set< std::string > key\_set() const

**Definition:** ast\_def.cpp:380

stan::gm::integrate\_ode::system\_function\_name\_

std::string system\_function\_name\_

**Definition:** ast.hpp:333

stan::gm::reject\_statement::reject\_statement

reject\_statement()

**Definition:** ast\_def.cpp:1335

stan::gm::positive\_ordered\_var\_decl::positive\_ordered\_var\_decl

positive\_ordered\_var\_decl()

**Definition:** ast\_def.cpp:1073

stan::gm::corr\_matrix\_var\_decl::corr\_matrix\_var\_decl

corr\_matrix\_var\_decl()

**Definition:** ast\_def.cpp:1149

stan::gm::int\_var\_decl

**Definition:** ast.hpp:455

stan::gm::contains\_var::contains\_var

contains\_var(const variable\_map &var\_map)

**Definition:** ast\_def.cpp:597

stan::gm::double\_literal::type\_

expr\_type type\_

**Definition:** ast.hpp:309

stan::gm::variable\_map::get\_base\_type

base\_expr\_type get\_base\_type(const std::string &name) const

**Definition:** ast\_def.cpp:996

stan::gm::contains\_nonparam\_var::operator()

bool operator()(const nil &e) const

**Definition:** ast\_def.cpp:684

stan::gm::transformed\_data\_origin

const int transformed\_data\_origin

**Definition:** ast.hpp:414

stan::gm::base\_expr\_type

int base\_expr\_type

**Definition:** ast.hpp:60

stan::gm::is\_nil

bool is\_nil(const expression &e)

**Definition:** ast\_def.cpp:768

stan::gm::statement::statement\_

statement\_t statement\_

**Definition:** ast.hpp:650

stan::math::e

double e()

Return the base of the natural logarithm.

**Definition:** constants.hpp:86

stan::gm::var\_decl::decl\_

var\_decl\_t decl\_

**Definition:** ast.hpp:609

stan::gm::fun

**Definition:** ast.hpp:350

stan::gm::printable::printable

printable()

**Definition:** ast\_def.cpp:589

stan::gm::array\_literal::type\_

expr\_type type\_

**Definition:** ast.hpp:317

stan::gm::for\_statement::variable\_

std::string variable\_

**Definition:** ast.hpp:719

stan::gm::expr\_type::operator!=

bool operator!=(const expr\_type &et) const

**Definition:** ast\_def.cpp:70

stan::gm::program::function\_decl\_defs\_

std::vector< function\_decl\_def > function\_decl\_defs\_

**Definition:** ast.hpp:796

stan::gm::sample::truncation\_

range truncation\_

**Definition:** ast.hpp:821

stan::gm::reject\_statement::printables\_

std::vector< printable > printables\_

**Definition:** ast.hpp:752

stan::gm::expr\_type::is\_ill\_formed

bool is\_ill\_formed() const

**Definition:** ast\_def.cpp:105

stan::gm::unit\_vector\_var\_decl

**Definition:** ast.hpp:472

stan::gm::fun::fun

fun()

**Definition:** ast\_def.cpp:853

stan::gm::function\_decl\_def::return\_type\_

expr\_type return\_type\_

**Definition:** ast.hpp:782

stan::gm::returns\_type\_vis

**Definition:** ast.hpp:688

stan::gm::name\_vis::name\_vis

name\_vis()

**Definition:** ast\_def.cpp:1160

stan::gm::binary\_op

**Definition:** ast.hpp:382

stan::gm::arg\_decl::base\_variable\_declaration

base\_var\_decl base\_variable\_declaration()

**Definition:** ast\_def.cpp:403

stan::gm::range::has\_high

bool has\_high() const

**Definition:** ast\_def.cpp:940

stan::gm::is\_assignable

bool is\_assignable(const expr\_type &l\_type, const expr\_type &r\_type, const std::string &failure\_message, std::ostream &error\_msgs)

**Definition:** ast\_def.cpp:1453

stan::gm::infer\_type\_indexing

expr\_type infer\_type\_indexing(const base\_expr\_type &expr\_base\_type, size\_t num\_expr\_dims, size\_t num\_index\_dims)

**Definition:** ast\_def.cpp:873

stan::gm::is\_nil\_op::operator()

bool operator()(const nil &x) const

**Definition:** ast\_def.cpp:754

stan::gm::function\_argument\_origin\_lp

const int function\_argument\_origin\_lp

**Definition:** ast.hpp:420

stan::gm::range::range

range()

**Definition:** ast\_def.cpp:931

stan::gm::INT\_T

const int INT\_T

**Definition:** ast.hpp:65

stan::gm::assignment::var\_type\_

base\_var\_decl var\_type\_

**Definition:** ast.hpp:831

stan::gm::expr\_type::operator>

bool operator>(const expr\_type &et) const

**Definition:** ast\_def.cpp:83

stan::gm::unary\_op::unary\_op

unary\_op(char op, expression const &subject)

**Definition:** ast\_def.cpp:923

stan::gm::index\_op::infer\_type

void infer\_type()

**Definition:** ast\_def.cpp:907

stan::gm::return\_statement

**Definition:** ast.hpp:757

stan::gm::expression::operator+=

expression & operator+=(const expression &rhs)

**Definition:** ast\_def.cpp:1386

stan::gm::variable

**Definition:** ast.hpp:323

stan::gm::binary\_op::binary\_op

binary\_op()

**Definition:** ast\_def.cpp:911

stan::gm::expression\_type\_vis::operator()

expr\_type operator()(const nil &e) const

**Definition:** ast\_def.cpp:533

stan::gm::function\_decl\_defs::decl\_defs\_

std::vector< function\_decl\_def > decl\_defs\_

**Definition:** ast.hpp:791

stan::gm::transformed\_parameter\_origin

const int transformed\_parameter\_origin

**Definition:** ast.hpp:416

stan::gm::expr\_type::operator<

bool operator<(const expr\_type &et) const

**Definition:** ast\_def.cpp:73

stan::gm::cholesky\_factor\_var\_decl::cholesky\_factor\_var\_decl

cholesky\_factor\_var\_decl()

**Definition:** ast\_def.cpp:1118

stan::gm::is\_no\_op\_statement\_vis

**Definition:** ast.hpp:671

stan::gm::distribution::args\_

std::vector< expression > args\_

**Definition:** ast.hpp:196

stan::gm::statements::statements

statements()

**Definition:** ast\_def.cpp:526

stan::gm::expr\_type::operator<=

bool operator<=(const expr\_type &et) const

**Definition:** ast\_def.cpp:78

stan::gm::double\_literal::operator=

double\_literal & operator=(const double\_literal &dl)

**Definition:** ast\_def.cpp:806

stan::gm::variable\_map::range\_t

std::pair< base\_var\_decl, var\_origin > range\_t

**Definition:** ast.hpp:440

stan::gm::sample::dist\_

distribution dist\_

**Definition:** ast.hpp:820

stan::gm::vector\_var\_decl

**Definition:** ast.hpp:504

stan::gm::name\_vis::operator()

std::string operator()(const nil &x) const

**Definition:** ast\_def.cpp:1161

stan::gm::function\_decl\_def::body\_

statement body\_

**Definition:** ast.hpp:785

stan::gm::function\_signatures::get\_result\_type

expr\_type get\_result\_type(const std::string &name, const std::vector< expr\_type > &args, std::ostream &error\_msgs)

**Definition:** ast\_def.cpp:329

stan::gm::while\_statement

**Definition:** ast.hpp:737

stan::gm::unary\_op::op

char op

**Definition:** ast.hpp:394

stan::gm::unit\_vector\_var\_decl::unit\_vector\_var\_decl

unit\_vector\_var\_decl()

**Definition:** ast\_def.cpp:1040

stan::gm::variable::name\_

std::string name\_

**Definition:** ast.hpp:324

stan::math::dist

double dist(const std::vector< double > &x, const std::vector< double > &y)

**Definition:** dist.hpp:11

stan::gm::int\_literal::val\_

int val\_

**Definition:** ast.hpp:298

stan::gm::variable\_map::exists

bool exists(const std::string &name) const

**Definition:** ast\_def.cpp:988

stan::gm::write\_base\_expr\_type

std::ostream & write\_base\_expr\_type(std::ostream &o, base\_expr\_type type)

**Definition:** ast\_def.cpp:23

stan::gm::statements::local\_decl\_

std::vector< var\_decl > local\_decl\_

**Definition:** ast.hpp:186

stan::gm::print\_statement::printables\_

std::vector< printable > printables\_

**Definition:** ast.hpp:746

stan::gm::expression::expression\_type

expr\_type expression\_type() const

**Definition:** ast\_def.cpp:570

stan::gm::var\_decl::var\_decl\_t

boost::variant< boost::recursive\_wrapper< nil >, boost::recursive\_wrapper< int\_var\_decl >, boost::recursive\_wrapper< double\_var\_decl >, boost::recursive\_wrapper< vector\_var\_decl >, boost::recursive\_wrapper< row\_vector\_var\_decl >, boost::recursive\_wrapper< matrix\_var\_decl >, boost::recursive\_wrapper< simplex\_var\_decl >, boost::recursive\_wrapper< unit\_vector\_var\_decl >, boost::recursive\_wrapper< ordered\_var\_decl >, boost::recursive\_wrapper< positive\_ordered\_var\_decl >, boost::recursive\_wrapper< cholesky\_factor\_var\_decl >, boost::recursive\_wrapper< cholesky\_corr\_var\_decl >, boost::recursive\_wrapper< cov\_matrix\_var\_decl >, boost::recursive\_wrapper< corr\_matrix\_var\_decl > > var\_decl\_t

**Definition:** ast.hpp:607

stan::gm::conditional\_statement::conditional\_statement

conditional\_statement()

**Definition:** ast\_def.cpp:1315

stan::gm::matrix\_var\_decl::M\_

expression M\_

**Definition:** ast.hpp:526

stan::gm::returns\_type

bool returns\_type(const expr\_type &return\_type, const statement &statement, std::ostream &error\_msgs)

**Definition:** ast\_def.cpp:515

stan::gm::variable::set\_type

void set\_type(const base\_expr\_type &base\_type, size\_t num\_dims)

**Definition:** ast\_def.cpp:829

stan::gm::contains\_nonparam\_var::var\_map\_

const variable\_map & var\_map\_

**Definition:** ast.hpp:870

stan::gm::int\_literal::operator=

int\_literal & operator=(const int\_literal &il)

**Definition:** ast\_def.cpp:792

stan::gm::arg\_decl::arg\_type\_

expr\_type arg\_type\_

**Definition:** ast.hpp:768

stan::gm::var\_origin

int var\_origin

**Definition:** ast.hpp:411

stan::gm::returns\_type\_vis::operator()

bool operator()(const nil &st) const

**Definition:** ast\_def.cpp:433

stan::gm::function\_signatures::add\_ternary

void add\_ternary(const ::std::string &name)

**Definition:** ast\_def.cpp:282

stan::gm::statements::statements\_

std::vector< statement > statements\_

**Definition:** ast.hpp:187

stan::gm::expr\_type::is\_primitive

bool is\_primitive() const

**Definition:** ast\_def.cpp:93

stan::gm::int\_var\_decl::int\_var\_decl

int\_var\_decl()

**Definition:** ast\_def.cpp:1016

stan::gm::positive\_ordered\_var\_decl::K\_

expression K\_

**Definition:** ast.hpp:497

stan::gm::statement::statement

statement()

**Definition:** ast\_def.cpp:1228

stan::gm::variable\_map::get\_origin

var\_origin get\_origin(const std::string &name) const

**Definition:** ast\_def.cpp:1002

stan::gm::contains\_var::var\_map\_

const variable\_map & var\_map\_

**Definition:** ast.hpp:851

stan::gm::return\_statement::return\_statement

return\_statement()

**Definition:** ast\_def.cpp:1324

stan::gm::index\_op

**Definition:** ast.hpp:370

stan::gm::ILL\_FORMED\_T

const int ILL\_FORMED\_T

**Definition:** ast.hpp:70

stan::gm::expr\_type::is\_void

bool is\_void() const

**Definition:** ast\_def.cpp:108

stan::gm::VOID\_T

const int VOID\_T

**Definition:** ast.hpp:64

stan::gm::for\_statement::for\_statement

for\_statement()

**Definition:** ast\_def.cpp:1297

stan::gm::while\_statement::while\_statement

while\_statement()

**Definition:** ast\_def.cpp:1307

stan::gm::is\_no\_op\_statement\_vis::operator()

bool operator()(const nil &st) const

**Definition:** ast\_def.cpp:1246

stan::gm::function\_signature\_t

std::pair< expr\_type, std::vector< expr\_type > > function\_signature\_t

**Definition:** ast.hpp:104

stan::gm::arg\_decl

**Definition:** ast.hpp:767


---

[ Stan Home Page ]

*© 2011–2014,
Stan Development Team.*
